# Thoracic aorta pseudoaneurysm with hemopericardium: unusual presentation of warfarin overdose

**DOI:** 10.1186/1745-6673-6-12

**Published:** 2011-04-26

**Authors:** Ya-Chih Tien, Ying-Cheng Chen, Chiung-Ying Liao, Chia-Chu Chang

**Affiliations:** 1 Department of Medicine, Changhua Christian Hospital, 135 Nan-Siau Street, Changhua city, 500 Taiwan; 2 Department of Cardiovascular Surgery, Changhua Christian Hospital, 135 Nan-Siau Street, Changhua city, 500 Taiwan; 3 Department of Radiology, Changhua Christian Hospital, 135 Nan-Siau Street, Changhua city, 500 Taiwan

**Keywords:** Warfarin, pseudoaneurysm, hemopericardium, TEVAR

## Abstract

There have been few case reports which discuss a relationship between warfarin overdose and aortic pseudoaneurysm leakage. We report the case of a female receiving warfarin who presented with dsypnea. Her international normalized ratio was > 10. Chest radiograph revealed cardiomegaly, and chest computed tomography (CT) showed a bulging pouch-like lesion below the aortic arch greater than 6x6 cm in size and a fluid collection suggesting blood in the pericardium. Thoracic endovascular aneurysm repair (TEVAR) was successfully performed by a cardiovascular surgeon. Aortic pseudoaneurysm formation and leakage may be considered as a rare complication in patients receiving warfarin therapy. Further study regarding warfarin use and the incidence of pseudoaneurysm leakage is needed.

## Background

A patient with a pseudoaneurysm will typically have had a traumatic event such as a recent blunt or penetrating trauma, or an endovascular procedure[1,2]. Heart failure and chest pain are the most common manifestations of a pseudoaneurysm of the ascending aorta[3]. Herein we report the case of a female receiving warfarin whose international normalized ratio (INR) was >10, who presented with dyspnea. Chest computed tomography (CT) revealed an aortic arch pseudoaneurysm and a fluid collection suggesting blood in the pericardium. We discuss the risk of bleeding as it is related to warfarin overdose and pseudoaneurysm leakage.

## Case presentation

A 78-year-old female, presenting with progressive shortness of breath and general weakness was admitted to our hospital on March 15, 2010. She experienced palpitations and tachycardia, and mild chest tightness when palpitations occurred. Her history was significant for primary cancer of the appendix with ovarian metastases, and was status post a debunking operation in December of 2006, complicated by chronic right leg lymphedema. She had been taking warfarin as prescribed by the cardiovascular surgery department for deep vein thrombosis of the right leg.

On admission, her blood pressure was 148/96 mmHg, heart rate 114 beats/min, respiratory rate 26 breaths/min, and temperature 37.8°C. Laboratory studies revealed: white blood cell (WBC) count, 17200/uL (neutrophil-segment 89.1%); hemoglobin, 7.6 gm/dL; platelet count, 455000/uL; NT-proBNP, 6776 pg/mL; PT, 143s (INR >10); blood urea nitrogen (BUN), 33 mg/dL; creatinine, 0.77 mg/dL; Na 131 mmol/L; K 2.5, mmol/L; Ca 8.4 mg/dL; Mg, 2.4 mg/dL; and albumin 1.7 g/dL. The thyroid function tests were normal. Artery gas analysis showed hypoxia (pH, 7.4; PCO_2_, 36.9 mm Hg; PO_2_, 75.7 mm Hg; HCO_3_, 23.4 mmol/L; SaO_2_, 95%). The elevated PT and INR suggested warfarin overdose. We prescribed VitK_1 _1 ample per-12h and transfused frozen fresh plasma 12 units per-day. Three days later, the PT was normalized, 21s (INR2.0).

**Figure 1:**
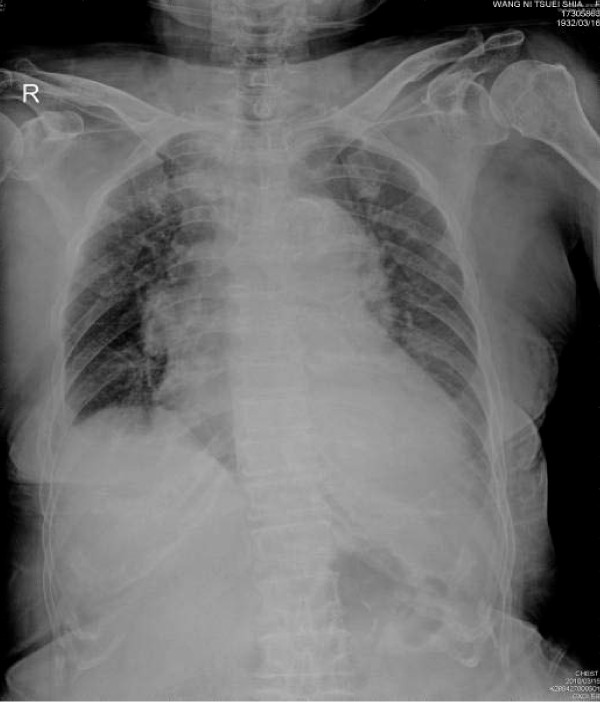
**Chest AP film on admission revealed cardiomegaly with widening of the mediastinum, as well as blunting of left costo-pleural angle suggesting pleural effusion**.

**Figure 2:**
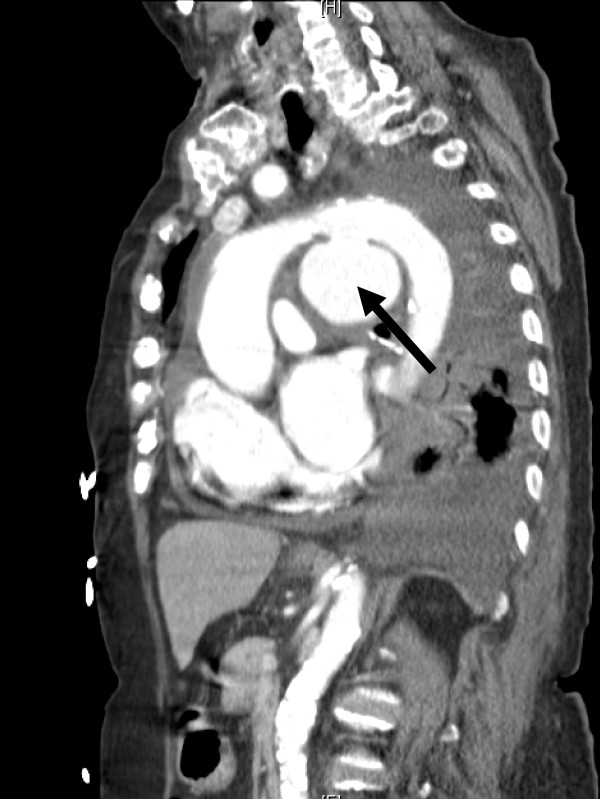
**Chest computed tomography (CT) in sagital oblique reformation:** a pseudoaneurysm size over 6*6 cm arises from aortic arch (black arrow) and suspicious hemorrhage into pericardium.

**Figure 3:**
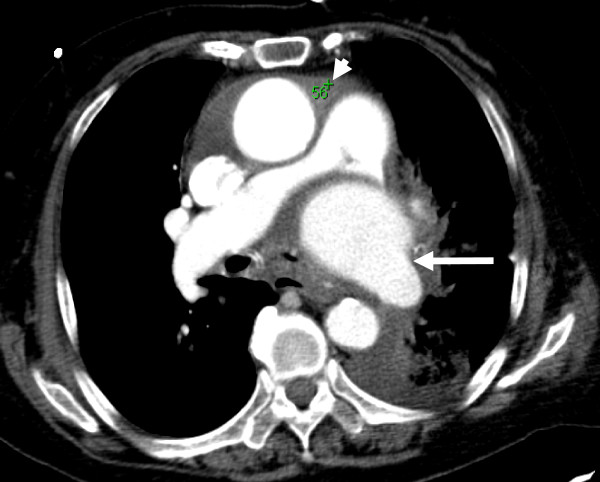
**Cross section of chest CT:** arrow (white) points the pseuoaneurysm, compression of pulmonary artery by pseudoaneuysm was noted. Pericardium effusion is identified in hyper-density (white arrow head) suggesting bloody component that may resulted from the pseudoaneurysm hemorrhage into pericardium space.

As admitted, her chest radiograph revealed cardiomegaly with pulmonary edema and blunting of the left costophrenic angle (Figure 1). Echocardiography revealed normal left ventricular systolic function with an ejection fraction of 70%, dilatation of the left atrium, right ventricle, and ascending aorta, moderate tricuspid valve regurgitation, mild pulmonary, mitral, and aortic valve regurgitation, and pericardial effusion; no valvular stenosis problem was identified. Chest CT was performed in consideration of an organic lesion, such as a pulmonary embolism or malignancy. A large bulging pouch-like lesion below the aortic arch greater than 6x6 cm in size and a fluid collection in the pericardium (relative high density) was found (Figure 2, 3). Results were consistent with a pseudoaneurysm in the aortic arch and hemorrhage into the pericardium.

**Figure 4:**
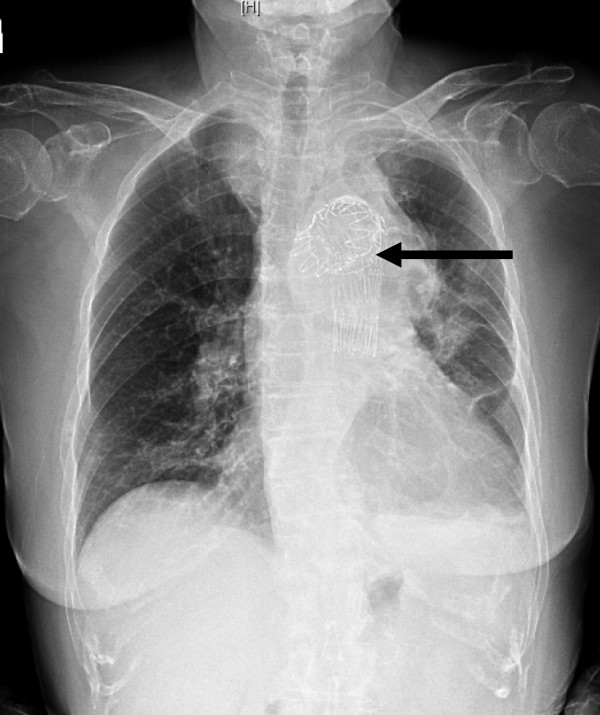
**Chest X ray: after thoracic endovascular aneurysm repair (black arrow point stent in aortic arch)**.

Thoracic endovascular aneurysm repair (TEVAR) was successfully performed by a cardiovascular surgeon one day later. Clinical presentation including serial CXR (Figure 4) and patient status showed dramatic improvement. The procedure was successful, and the patient was discharged 2 weeks later in good condition. At follow-up in the cardiovascular surgery department she remained in stable condition.

## Discussion

Etiologies of ascending aortic pseudoaneurysms include trauma, connective tissue disease, vasculitis, and prior aortic surgery[1,2]. Doppler ultrasound can detect pseudoaneurysm, and is inexpensive and widely available; however, CT, arteriography, and CT angiography are superior at showing the anatomy of the arterial system[4]. Once a pseudoaneurysm is diagnosed, endovascular management is the best treatment option[5].

Major bleeding has been reported in 1.1%-8.1% of patients during each year of long term warfarin therapy, and risk factors include old age, hypoalbuminemia, serious illness (cardiac, kidney, or liver disease), cerebrovascular or peripheral vascular disease, and an unstable anticoagulant effect[6]. This effect is related to warfarin being absorbed after oral administration, and then being highly bound to albumin in plasma[7]. Thus, hypoalbuminemia is associated with an increased risk of over-anticoagulation. One study showed that in patients on long term warfarin therapy, there was a 32% increase in all forms of bleeding, and a 46% increase in major bleeds for every 10 years of age over 40 years[8].

Blunt et al. reported a warfarin-associated thoracic aortic dissection in an elderly woman, and concluded that the mechanism of aortic dissection was a bleed into an atheromatous plaque in the thoracic aorta, which was related to warfarin therapy[7].

## Conclusion

Aortic aneurysm formation and leakage may be a rare complication in patients receiving warfarin therapy that has not been previously reported. Further study regarding warfarin use and the incidence of aneurysm leakage may be an interesting topic worthy of additional examination.

## Consent

Written informed consent was obtained from the patient for publication of this case report and accompanying images

## Competing interests

The authors declare that they have no competing interests.

## Authors' contributions

YCT contributed in visiting the case, all authors contributed in editing the manuscript, all authors contributed in drafting the manuscript, all authors read and approved the final manuscript.
